# Holter STAT-ON™ against other tools for detecting MF in advanced Parkinson’s disease: an observational study

**DOI:** 10.3389/fneur.2023.1249385

**Published:** 2023-08-18

**Authors:** Iria Cabo-Lopez, Alfredo Puy-Nuñez, Nuria Redondo-Rafales, Sara Teixeira Baltazar, Beatriz Calderón-Cruz

**Affiliations:** ^1^Neurology Department, University Hospital Complex of Pontevedra, Galicia, Spain; ^2^Methodology and Statistics Unit, Galicia Sur Health Research Institute, Galicia, Spain

**Keywords:** Parkinson disease, motor complications, machine learning, wearable electronic devices, levodopa

## Abstract

**Background:**

Different screening tools to identify advanced Parkinson’s disease (APD) have emerged in recent years. Among them, wearable medical devices, such as STAT-ON™, have been proposed to help to objectively detect APD.

**Objectives:**

To analyze the correlation between STAT-ON™ reports and other assessment tools to identify APD and to assess the accuracy of screening tools in APD patients, using the STAT-ON™ as the gold standard.

**Methods:**

In this retrospective, observational study, data from the University Hospital Complex of Pontevedra database on 44 patients with potential APD who wore STAT-ON™ were extracted. Data were collected according to different sources of tools for identifying APD: (1) STAT-ON™, (2) information provided by the patient, (3) questionnaire for advanced Parkinson’s disease (CDEPA), (4) 5-2-1 Criteria, and (5) Making Informed Decisions to Aid Timely Management of Parkinson’s Disease (MANAGE-PD). Considering STAT-ON™ recordings as a reference, the sensitivity, specificity, and positive and negative predictive values for each tool were calculated. The *kappa* index assessed the degree of agreement between the gold standard and the other instruments.

**Results:**

Although no statistically significant association was found between STAT-ON™ recordings and any screening methods evaluated, the CDEPA questionnaire demonstrated the highest sensitivity and VPN values to detect patients with APD candidates for second-line therapy (SLT). According to the correlation analyses, MANAGE-PD demonstrated the highest degree of concordance with STAT-ON™ recordings to identify the SLT indication and to predict the SLT decision.

**Conclusion:**

STAT-ON™ device may be a helpful tool to detect APD and to guide treatment decisions.

## 1. Introduction

Parkinson’s disease (PD) is a neurodegenerative disorder characterized by cardinal motor symptoms (bradykinesia, resting tremor, rigidity, and postural instability) as well as a wide range of non-motor complications (e.g., cognitive impairment, mental health disorders, and sleep disorders) (1). Such changes in PD frequently limit functional independence and are the leading cause of morbidity and mortality among PD patients (2).

In the early stages, dopaminergic treatment has been demonstrated to improve motor symptoms and quality of life in PD patients (3). However, with time, treatment efficacy decreases and motor complications such as motor fluctuations (MF) and dyskinesias arise, which may interfere with the patient’s activity (4). Apart from the motor symptoms non-MF are also present, which can thus have a significant impact on the person’s quality of life (5, 6).

Despite all therapeutic adjustment efforts, 90% of patients experience motor complications after 10 years (7), which are common in the early stages of PD and are underestimated by routine neurological clinical evaluation (8). Motor complications can be variable in character, fluctuating between days and even throughout the day. Therefore, the pattern in symptom chronology is of great value for the precise adjustment of medication dosage (8, 9). As such, it is relevant to determine the patient’s clinical characteristics that can define advanced Parkinson’s disease (APD) and make them eligible for advanced therapies. However, due to the fluctuating and irregular nature of motor manifestations, such information is hard to collect in routine practice. Additionally, a lack of consensus around the definition of advanced disease leads to delays in the identification of advancing PD, resulting in heterogeneity of care. This compounds the challenges of managing disease progression and timely treatment (10). In clinical practice, adjusting symptomatic treatment to improve a patient’s quality of life and autonomy requires a simple tool to identify patients in the more advanced stages of PD. In this context, in recent years, several instruments have been proposed to facilitate the timely identification and management of patients with advancing PD with suboptimal symptom control while on standard therapy, such as diaries (e.g., Hauser diary) (11), questionnaires [e.g., the questionnaire for advanced Parkinson’s disease—*Cuestionario de enfermedad de Parkinson avanzada*—(CDEPA)] (12), educational programs (e.g., Navigate-PD) (13), consensus clinical criteria (e.g., 5-2-1 Criteria) (14), and clinician-reported tools (e.g., the Making Informed Decisions to Aid Timely Management of Parkinson’s Disease —MANAGE-PD—) (15).

However, while the latter methods continue to be the reference standard in PD research and care, they have serious limitations since the subjectivity and cognitive state of patients greatly impacts upon the reliability of the results, and few can adhere to such laborious systems beyond several days (11). Additionally, these tools are limitated in terms of the quality of the information collected due to memory bias, as discontinuous monitoring via clinical visits and in-person assessments does not capture PD symptoms and their progression completely. Indeed, patients’ self-reported data of their improvement over time in response to treatment do not agree with their UPDRS scores from in-person appointments (16). Thus, a new system objective, capable of automatically and continuously detecting and recording MF and being part of the patient’s day-to-day management long-term, might be of great utility in clinical practice to help to optimize medication regimens and improve disease control (17). Recently, new artificial intelligence-based systems are emerging to detect and quantify motor symptoms in PD patients. There are multiple research projects in progress that aim to on improve the identification of motor symptoms, wherein accelerometers are the most widely used sensors. However, gyroscopes, electromyography, skin conductivity, systems pressure insoles, and pressure platforms are also used. STAT-ON™ is an inertial wearable medical device Class IIa that is able to monitor, measure, hold in internal memory, and generate a report on the temporal evolution of motor symptoms in daily living conditions. The STAT-ON™ system consists of a monitoring device, a base charger, a belt, and a mobile application. The sensor is held in place by a custom-designed strap that conforms to the body of each user. Once clinicians configure the system, STAT-ON™ provides numerical and graphical information about the motor symptoms’ presence and distribution associated with PD, based on a real-time processing embedded version of specific algorithms. The utility and acceptability of STAT-ON™ in real clinical practice are promising (18). Indeed, STAT-ON™ has achieved excellent results in the detection or characterization of MF, dyskinesia, and gait freeze and is, hence, of significant help to optimize treatment in APD patients eligible for second-line therapies (SLT) (19). This study aimed to analyze the correlation between STAT-ON™ reports and other motor assessment tools for MF and to assess the accuracy of screening tools in APD patients, using the STAT-ON™ device recordings as the gold standard.

## 2. Materials and methods

This was a single-center, retrospective, observational study in which each participant wore the STAT-ON™ device under real-life conditions. This study has been approved by the appropriate ethical committees related to the institution (The Galician Network of Research Ethics Committees).

### 2.1. Participants

Data were used from the Movement Disorders monographic consultation database at the University Hospital Complex of Pontevedra, including pseudo-anonymized information on PD patients who wore STAT-ON™ from November 4, 2019 to March 31, 2022. Forty-four PD patients were included in this study to compare the efficiency of STAT-ON™ against classical clinical practice methods in terms of APD detection. PD patients were recruited according to specific inclusion criteria: diagnosis of idiopathic PD based on current standards in patients with suspected APD with the indication of use for STAT-ON™ Holter on active mode. No exclusion criteria were established. The study was conducted according to the guidelines of the Declaration of Helsinki, and demographic and clinical data were noted anonymously.

### 2.2. Data analyses and outcome measures

Only the minimum information necessary to achieve the purposes of this study was collected and analyzed, taking into account the principle of data minimization established by the General Data Protection Regulation (Directive (EU) 2016/680 of the European Parliament and of the Council of 27 April 2016), articles 5 and 89. The information collected came from the database “Movement Disorders Monographic Consultation,” which includes the following variables: month/year of birth, gender, PD diagnosis date, number of daily doses of levodopa, data regarding the clinical scales [Movement Disorders Society-Unified Parkinson’s disease rating scale—UPDRS-III— (20), Hoehn & Yahr scale—H&Y—] (21), PD motor symptoms (ON and OFF periods, ON time with disabling dyskinesias, unpredictable fluctuations, freezing of gait, painful dystonia, dysphagia, dysarthria, balance disturbance, and falls), non-motor symptoms (dysautonomia, daytime sleepiness, cognitive impairment/dementia, hallucinations with/without insight, apathy, psychotic symptoms, and impulse control disorders), and the degree of disability. Data were collected from PD patients according to different sources of information: (a) STAT-ON™ Holter; (b) information provided by the patient during the visit; (c) MANAGE-PD, a designed tool to aid clinicians in determining which PD patients may not be adequately controlled on their current treatment regimen and may require second-line therapy (SLT); (d) 5-2-1 Criteria, an instrument to identify APD patients who require optimization of their Parkinson’s treatment; and (e) CDEPA questionnaire, a screening tool for the early diagnosis of APD. All parameters were assessed at the same time.

In contrast to the objective data about the daily OFF-time collected by STAT-ON™, the information regarding the daily OFF-time required to complete these tools (Criteria 5-2-1, CDEPA, and MANAGE-PD) was obtained from the patient, based on their subjective perception of their daily OFF-time. The time spent in intermediate and indeterminate states was not taken into account when calculating daily OFF-time. When a patient presented with ≥2 h of OFF-time/day (detected by STAT-ON™, 5-2-1 criteria, or the patient’s self-perception), APD was considered as a subsidiary of receiving SLT. In order to assess the accuracy of the examined methods, sensitivity, specificity, positive predictive value (PPV), and negative predictive value (NPV) were calculated for the mean number of hours spent in the OFF-motor phase obtained, using STAT-ON™ results as a reference. In addition, the extent of agreement between the gold standard and the other instruments was assessed by the *kappa* index.

### 2.3. Statistical analyses

Data were analyzed with the statistical package SPSS v. 25.0. The qualitative variables were analyzed using absolute frequencies and percentages. Quantitative variables were displayed as the mean ± standard deviation (SD), or as median and interquartile ranges, according to their distribution. The Shapiro–Wilk test was used to verify whether the data followed a normal distribution. The Fisher or Chi-square test was used to determine the association between the qualitative variables. Additionally, Cohen’s Kappa was calculated in order to estimate the concordance between the variables and performed analyses of sensitivity, specificity, and positive and negative predictive values. Values of *p* < 0.05 were considered to be statistically significant.

### 2.4. Data sharing

The data supporting this study’s findings are available from the corresponding author upon reasonable request.

## 3. Results

### 3.1. Baseline data of participants

Forty-four PD patients underwent a motor assessment with a STAT-ON™ device during a mean of 70.2 h (range, 30–119). At the time of STAT-ON™ placement, data on the UPDRS-III score and H&Y stage could be obtained in 29 of 44 patients (65.9%) and 39 of 44 patients (88.6%), respectively. Baseline characteristics of included PD patients are displayed in Table 1.

**Table 1:** Characteristics of the participants analyzed in this study.

Number of subjects (*n*)	44
Age (years)	66.3 ± 9.0 (43–81)
Gender (M: F)	20:24
Disease duration (years)	8.1 ± 4.1 (0–20)
UPDRS-III scores at the placement of STAT-ON™	22.5 ± 11.0 (4–51)
Hoehn and Yahr Stage at the placement of STAT-ON™	2.4 ± 0.6 (1–4)
STAT-ON™ time (hours)	70.2 ± 15.6 (30–119)
Levodopa daily dosing frequency	4.6 ± 1.1 (3–8)

The results represent mean ± SD (ranges). Data about H&Y and UPDRS-III are during the OFF and ON states. UPDRS, Unified Parkinson’s Disease Rating Scale; SD, Standard deviation.

STAT-ON™ detected MF in 42 of the 44 PD patients (95.4%) who wore this Holter device due to suspicion of motor complications. The mean duration of OFF-time recorded by the STAT-ON™ device was 2.9 h (SD: 1.57; range 0.0–6.7 h), corresponding to the motion records of the 44 subjects. For 33 patients (75%) included in this study, the mean time spent on the OFF motor state was ≥2 h per day, while for the remaining 11 patients (25%), this mean duration was <2 h per day.

Two patients self-reported MF, but STAT-ON™ was unable to corroborate them. The total monitoring period for the first patient (a 45-year-old female with H&Y stage 2) was 48 hours (significantly below average). She perceived MF either when the STAT-ON™ reports indicated an intermediate state (between ON and OFF states) or when the Holter monitor had yet to be placed during the earliest hours of the day. Because the sensor had not yet been put into place, we believe that STAT-ON™ did not detect morning akinesia. In the second patient, a 78-year-old woman, the total monitoring duration was 70 hours. When the STAT-ON™ device was put on the patient, she was clearly undertreated with significant difficulty walking (H&Y 4-5 stage), and we suspected she had a clear APD. 98% of STAT-ON™ reports exhibited an indeterminate state, indicating that the sensor was unable to detect any movement. Therefore, we consider that it is highly likely that the patient had such a severe and continuous OFF time that she could hardly move, and indeterminate states detected corresponded with a state in OFF.

Regarding other common motor complications in APD patients, 23 of the 44 PD patients (52.2%) self-reported freezing of gait episodes: 14 of them (60.8%) were also reported by STAT-ON™, while the device did not detect 9. Of the 16 patients classified by the Holter as freezing of gait episodes, only 2 (12.5%) were not consistent with patient self-assessment reports. Among the 44 included patients, 8 participants (18.1%) self-reported disabling dyskinesias.

### 3.2. APD detection

#### 3.2.1. STAT-ON™ vs. patient self-reported data

Of the 17 patients reporting non-significant MF in OFF (mean duration <2 h per day), only three patients (17.6%) STAT-ON™ recorded the same information regarding the mean hours on OFF, while for the remaining 14 (82.4%) patients, the mean time on OFF was ≥2 h per day. Of the 27 patients who reported the presence of significant MF on OFF (≥ 2 h per day), in 19 (70.4%) patients, the STAT-ON™ reports coincided with the mean duration reported by the patient (i.e., ≥ 2 h). Conversely, STAT-ON™ recorded a mean OFF-time of <2 h per day in eight patients (29.6%), unlike the 19 patients reporting a mean of ≥2 h spent on OFF-time. In the 6 patients with a mean OFF-time of <2h per day according to STAT-ON™ reports, the mean duration of OFF periods was 1.3 h (range: 0.3-1.9). Although Cohen’s Kappa test indicated a slight disagreement between the STAT-ON™ recordings and the MF reported by patients, the result was not statistically significant (Kappa = −0.12; *p* = 0.371). Furthermore, Fisher’s exact test did not reveal an association between the variables obtained with both methods (*p* = 0.486). Thus, considering STAT-ON™ readings as the gold standard, the accuracy values for detecting ≥2 h per day of MF in OFF state by the patient were as follows: sensitivity 0.57, specificity 0.27, PPV 0.70, and NPV 0.17.

#### 3.2.2. STAT-ON™ vs. MANAGE-PD tool

Making Informed Decisions to Aid Timely Management of Parkinson’s Disease is a digital tool that facilitates informed decision-making to support the timely management of PD, by identifying which patients appear to be adequately controlled on their current treatment regimen versus which patients likely require treatment adjustment. In this study, 34 patients (77.3%) were classified as candidates to change their treatment regimen to SLT using MANAGE-PD, while 33 (75.0%) were considered candidates using the STAT-ON™ device. Again, Cohen’s Kappa test indicated a slight disagreement between the STAT-ON™ and the MANAGE-PD tool results, but this difference was not statistically significant (Kappa = −0.18; *p* = 0.213). When STAT-ON™ recordings were defined as the gold standard, the accuracy values for detecting APD patients candidates for SLT by MANAGE-PD tool were: sensitivity 0.72, specificity 0.09, PPV 0.70, and NPV 0.10.

#### 3.2.3. STAT-ON™ vs. 5-2-1 criteria tool

The results were analyzed between the 5- (five times oral levodopa tablet taken/day) 2- (2 h of OFF-time/day) 1 (1 h/day of troublesome dyskinesia) criteria screening tool to identify APD patients who require optimization of PD treatment. Thirty-six patients (81.8%) were 5-2-1 criteria positive (defined as meeting ≥1 of the criteria), of which only 26 (72.2%) were considered candidates according to STAT-ON™ results. However, this minor disagreement between both methods was not statistically significant in Cohen’s Kappa test (Kappa = −0.133, *p* = 0.367). Based on these results, the accuracy values for detecting APD patients candidates for SLT by the 5-2-1 Criteria tool were: sensitivity 0.78, specificity 0.09, PPV 0.72, and NPV 0.12.

#### 3.2.4. STAT-ON™ vs. CDEPA questionnaire

Cuestionario de enfermedad de Parkinson avanzada is a simple screening tool to identify patients with APD in the clinical setting based on the presence of any definitive symptom, which includes the presence of MF. According to this questionnaire, 38 PD patients (86.3%) were classified as candidates to change their treatment regimen, of whom 28 (73.6%) were also detected using the STAT-ON™ device. Although Cohen’s Kappa test demonstrated minor disagreement between the STAT-ON™ recordings and the answers to the CDEPA questionnaire, this result was not statistically significant (Kappa = −0.07; *p* = 0.612). Thus, the accuracy values for detecting APD patient candidates for SLT by CDEPA questionnaire were as follows: sensitivity 0.84, specificity 0.09, PPV 0.73, and NPV 0.16. Table 2 summarizes the accuracy values of sensitivity, specificity, PPV, and NPV for all analyzed tools.

**Table 2:** Sensitivity, specificity, PPV, and NPV for the different screening tools for MF in advanced EP patients.

	Patient self-reported data	MANAGE-PD	5-2-1 criteria	CDEPA questionnaire
Sensitivity	57.58%	72.73%	78.79	**84.85**
Specificity	**27.27%**	9.09%	9.09	9.09
PPV	70.37%	70.59%	72.22	**73.68**
NPV	**17.65%**	10.00%	12.50	16.67

CDEPA, questionnaire for advanced Parkinson’s disease questionnaire—Cuestionario de enfermedad de Parkinson avanzada; MANAGE-PD, making informed decisions to aid timely management of Parkinson’s disease; NPV, negative predictive value; PPV, positive predictive value. The highest values for each accuracy metric are shown in bold.

### 3.3. Identification of indication for SLT

According to STAT-ON™ device records, 33 patients (75.0%) were classified as candidates for SLT, in comparison to 34 patients (77.2%) using MANAGE-PD, 36 patients (81.8%) using the 5-2-1 criteria, and 38 patients (86.3%) with the CDEPA questionnaire. Fisher’s exact test demonstrated no association between the results obtained by the STAT-ON™ device and the 5-2-1 criteria tool (*p* = 0.145) or the CEDPA questionnaire (*p* = 0.083). However, the analyses revealed a statistically significant association between the STAT-ON™ and MANAGE-PD tool results (*p* = 0.032). The strength of this association was weak to moderate, with a *phi* coefficient value of 0.394. That is, the STAT-ON™ conclusions were consistent with the MANAGE-PD tool to classify patients with APD as candidates for SLT. The accuracy values of the MANAGE-PD tool to detect the need for SLT indication were: sensitivity 0.30, specificity 0.97, PPV 0.75, and NPV 0.83.

### 3.4. Prediction of the decision on SLT

STAT-ON™ recorded significant MF (>2 h per day) in 14 patients who were unable to recognize this phenomenon. In these patients, the following therapeutic decisions were made: three patients received optimization of conventional therapy; five patients had a specific indication for SLT but did not initiate SLT; one patient received deep brain stimulation (DBS); two patients received subcutaneous apomorphine infusion; and three patients received levodopa intestinal gel infusion as part of SLT. In view of the results, a strong (*phi* coefficient: 0.73) correlation was found between the STAT-ON™ conclusions and the therapeutic decision made (*p* < 0.001). Thus, for predicting the subsequent treatment that patients received, the STAT-ON™ accuracy values were: sensitivity 1.00, specificity 0.93, PPV 0.57, and NPV 1.00. On the other hand, no correlation was found between the STAT-ON™ device results and the 5-2-1 criterion tool (*p* = 0.014) or the CEDPA questionnaire (*p* = 0.042). Nonetheless, there was a statistically significant association between the results of STAT-ON™ and the MANAGE-PD tool for predicting SLT among patients with APD (*p* = 0.004).

## 4. Discussion

The importance of automatic detection of motor status lies in providing accurate information for physicians to adjust medication schedules and the possibility of identifying APD patients who are uncontrolled by conventional therapy and are candidates for SLT (e.g., subcutaneous apomorphine infusion, duodenal infusion of levodopa, or deep brain stimulation). In this study, the accuracy of different screening tools for detecting MF and APD patients was assessed, using the STAT-ON™ motor assessment as the gold standard. Based on the results from 44 APD patients, there was no statistically significant association between STAT-ON™ recordings and any screening methods evaluated to detect APD patients. Although all evaluated approaches to detect progression to APD had optimal accuracy, the CDEPA questionnaire demonstrated the highest sensitivity and PPV values (0.84 and 0.73, respectively). In contrast, the patient self-reported data method achieved the highest specificity and NPV values (0.27 and 0.17, respectively). According to these findings, all analyzed screening tools demonstrated high sensitivity values, except for the information provided by the patient, which significantly underestimated the presence of MF. Underestimation of MF may result in inadequate medication control and may delay the initiation of SLT. However, while the CDEPA and MANAGE-PD tools collect more information than those specifically designed to detect MF alone (such as STAT-ON™, patient self-perception, and 5-2-1 criteria), they also require more time to complete, which may represent an important limitation to their use. Nevertheless, when STAT-ON™ is unavailable to clinicians, CDEPA and MANAGE-PD remain comprehensive and widely accessible alternatives. STAT-ON™ is an easy-to-use device for both the healthcare professional and the patient, as it requires little time for placement and removal and it provides a continuous record over several days, thereby getting closer to the actual motor situation of the patient.

Regarding the identification of candidates for SLT, no association was found between the STAT-ON™ results-based decision and the 5-2-1 criteria tool or the CEDPA questionnaire. However, a weak to moderate correlation was observed between the STAT-ON™ and MANAGE-PD tool results to classify patients with APD as candidates for SLT, with a sensitivity of 0.30, specificity of 0.97, PPV of 0.75, and NPV of 0.83. Concerning the selection of APD patients receiving SLT, a strong correlation was demonstrated between the STAT-ON™ conclusions and the therapeutic decisions made, showing high accuracy values for predicting the treatment received in the patient (sensitivity 1.00, specificity 0.93, PPV 0.57, and NPV 1.00). The STAT-ON™ device results were not correlated with the 5-2-1 criteria tool or the CEDPA questionnaire, but they were statistically significant with the MANAGE-PD tool to predict SLT in APD patients.

This study supports STAT-ON™ as a sensitive screening tool for SLT prediction. Indeed, nearly all PD patients classified as requiring SLT by this Holter device received SLT. Similar results were obtained in an Argentinian study (22), where STAT-ON™ was compared with the data provided by 11 participants. The Holter registers were found to be more substantial than diary monitors, which supported the use of the STAT-ON™ to guide therapeutic decisions in clinical practice, especially in APD patients needing SLT. This Holter device has also been validated previously in further studies, demonstrating a better detection of ON/OFF MF, dyskinesia, and falls against patients’ diaries (23), as well as supporting its use as a valuable complementary tool to assess PD motor complications and the need for treatment adjustments (24). Furthermore, the ongoing multicenter, randomized clinical trial MoMoPa-EC will investigate this issue by establishing three study arms (STAT-ON™ data vs. Hauser diaries vs. clinical data only collected during the visit) (25, 26).

This study has some limitations that should be considered while interpreting the results. Firstly, due to the absence of a single, consensus-based definition, ≥ 2 h of OFF-time per day was established as a criterion to consider a patient with advanced-stage PD eligible for SLT. However, while this criterion appears to be aligned with clinical practice, this statement cannot be applied to all PD patients, and some patients in earlier PD stages may have been misclassified with APD.

Secondly, the lack of a formal description of MF is a key observation made during this study. In order to facilitate accurate classification, a more precise definition of MF is required: ideally one that takes into account the magnitude of fluctuations. Thirdly, the reports of STAT-ON™ were used as a reference to assess the validity of other screening tools. However, it should be emphasized that, to date, a recognized gold standard to detect MF does not exist.

Finally, given this study’s design, its technological complexity, and the fact that it was conducted in a “free-living-environment,” the PD sample size was limited and heterogeneity could not be avoided. This limits the generalizability of these results to the wider population. Nevertheless, despite the limitations of this study, to the best of the authors´ knowledge, this is the first study carried out to evaluate the accuracy of several assessment tools to identify APD patients. Furthermore, for the first time, this study used STAT-ON™ recordings as the gold standard to determine the sensitivity, specificity, PPV, and NPV values of those screening tools. On the other hand, it is noted that the nature of motor complications can vary from day to day and throughout the day, even depending on the individual’s stage of Parkinson’s disease, making it difficult to identify and classify them. This limitation for the detection of MF is shared with any of the tools we use, including STAT-ONTM. However, unlike other instruments, STAT-ONTM allows data collection over multiple days, which is an advantage over other clinical tools. Nevertheless, it is noted that when the patient does not use the device during all the specified hours, the STAT-ONTM report may be misleading (it could underestimate the OFF hours). In addition, patients in very advanced stages with limited mobility are not good candidates for recording OFF-times using STAT-ONTM due to the sensor must detect movement for an accurate register.

It should be emphasized that wearable medical devices, such as STAT-ON™, represent a system to monitor symptoms in a patient’s daily life, facilitating the objective evaluation of MF presence by professional raters. Further research, such as the MoMoPa-EC mentioned above, will investigate whether automated symptom monitoring systems improve the clinical control of PD patients with MF. The authors postulate that conducting additional studies is worthwhile to evaluate the validity of wearable systems with larger samples and longer monitoring times. Given the increasing interest in this research topic, if additional studies support this study’s preliminary findings, the STAT-ON™ device could be an alternative tool to patient diaries, with the benefits that they appear to be more objective and reliable and that they enhance patient empowerment.

In conclusion, the results of this study support the high level of sensitivity (which is the most critical characteristic of a screening tool) of the STAT-ON™ device to identify APD patients requiring SLT. Because this device was able to detect almost all (95.4%) analyzed PD patients with suspicion of MF and at least 70% of significant MF (>2 h/day) in PD patients, the STAT-ON™ device represents a useful tool to guide treatment decisions in routine clinical practice. The correlation analyses between the available screening tools (patient self-reported data, MANAGE-PD, 5-2-1 criteria, and the CDEPA questionnaire) and the STAT-ON™ recordings demonstrate that MANAGE-PD presents the highest degree of concordance, both to identify the SLT indication and to predict the SLT decision.

Thus, according to these results, the use of STAT-ON™ in PD patients to monitor MF might offer an objective measure of the APD patient’s motor state, provide additional value to PD neurologists, and guide treatment decisions. Furthermore, the implementation of such systems in routine clinical practice could allow for more precise regulation of pharmaceutical therapy and enable patients to benefit earlier from SLT, such as continuous infusion pumps and deep brain stimulation (DBS).

## Data availability statement

The raw data supporting the conclusions of this article will be made available by the authors, without undue reservation.

## Ethics statement

The studies involving humans were approved by The Galician Network of Research Ethics Committees. The studies were conducted in accordance with the local legislation and institutional requirements. The participants provided their written informed consent to participate in this study.

## Author contributions

IC-L: design, execution, analysis, writing, and editing of the final version of the manuscript. AP-N and NR-R: design, execution, writing, and editing of the final version of the manuscript. ST and BC-C: statistical analysis. All authors contributed to the article and approved the submitted version.

## Funding

The medical writer for this manuscript was funded by AbbVie in the context of a contract of service. AbbVie did not select authors for participation in the manuscript. No payments were made to the authors for the development of this manuscript. The authors maintained complete control over the manuscript content, and it reflects their opinions. AbbVie did not review the final manuscript draft for scientific accuracy and was not involved in methodology, data collection, analysis, or drafting.

## Conflict of interest

The authors declare that the research was conducted in the absence of any commercial or financial relationships that could be construed as a potential conflict of interest.

## Publisher’s note

All claims expressed in this article are solely those of the authors and do not necessarily represent those of their affiliated organizations, or those of the publisher, the editors and the reviewers. Any product that may be evaluated in this article, or claim that may be made by its manufacturer, is not guaranteed or endorsed by the publisher.
